# A Data Cleaning Method for Big Trace Data Using Movement Consistency

**DOI:** 10.3390/s18030824

**Published:** 2018-03-09

**Authors:** Xue Yang, Luliang Tang, Xia Zhang, Qingquan Li

**Affiliations:** 1 State Key Laboratory of Information Engineering in Surveying, Mapping, and Remote Sensing, Wuhan University, Wuhan 430079, China; yangxue@whu.edu.cn; 2 School of Urban Design, Wuhan University, Wuhan 430070, China; xiazhang@whu.edu.cn; 3 College of Civil Engineering, Shenzhen University, Shenzhen 518060, China; liqq@szu.edu.cn

**Keywords:** data cleaning, big data, vehicle trajectory, movement consistency modeling

## Abstract

Given the popularization of GPS technologies, the massive amount of spatiotemporal GPS traces collected by vehicles are becoming a new kind of big data source for urban geographic information extraction. The growing volume of the dataset, however, creates processing and management difficulties, while the low quality generates uncertainties when investigating human activities. Based on the conception of the error distribution law and position accuracy of the GPS data, we propose in this paper a data cleaning method for this kind of spatial big data using movement consistency. First, a trajectory is partitioned into a set of sub-trajectories using the movement characteristic points. In this process, GPS points indicate that the motion status of the vehicle has transformed from one state into another, and are regarded as the movement characteristic points. Then, GPS data are cleaned based on the similarities of GPS points and the movement consistency model of the sub-trajectory. The movement consistency model is built using the random sample consensus algorithm based on the high spatial consistency of high-quality GPS data. The proposed method is evaluated based on extensive experiments, using GPS trajectories generated by a sample of vehicles over a 7-day period in Wuhan city, China. The results show the effectiveness and efficiency of the proposed method.

## 1. Introduction

Nowadays, big data are everywhere, from sensors that monitor traffic loads to the flood of tweets and Facebook ‘likes’. Researchers use volume, velocity, variety, value, and veracity to characterize the key properties of those big data [1]. In contrast to volume, velocity, variety, and value, the fifth ‘V’ of big data, veracity, is increasingly recognized as a key dimension when making big data operational in various applications [2]. Big GPS trace data generated by vehicles also have the five ‘V’ characteristics [3] and provide us with an unprecedented window into the dynamics of urban areas [4,5,6,7,8,9]. However, the growing volume of spatial data brings significant challenges for the management of data processing. In addition, a large amount of low-quality data mixed in the raw dataset increases the uncertainty of knowledge mining. Therefore, data cleaning plays a crucial role in the research field of information science [10,11,12].

In this paper, we propose an efficient method for big trace data cleaning. On the basis of our previous work [12], the proposed method polishes the theory of GPS data cleaning with further development. To keep the consistency of moving objects, the entire trajectory is first partitioned into a set of sub-trajectories by movement characteristic points. Those characteristic points are extracted from trajectories based on the changes in the motion status. Then, GPS data are cleaned based on the similarities of GPS points and the movement consistency model of the sub-trajectory. The movement consistency model is built using the random sample consensus algorithm based on the high spatial consistency of high-quality GPS data. Moreover, the accuracy of cleaned data can be controlled by tuning the threshold of similarities of GPS data and the movement consistency model. The proposed method is evaluated based on extensive experiments, using GPS trajectories generated by a sample of vehicles over a 7-day period in Wuhan city, China. The results show the effectiveness and efficiency of the proposed method. In summary, the contributions of this research include: (1) High-quality GPS data can be extracted from the raw dataset using the proposed data cleaning method; (2) The method of vehicle movement consistency modeling is proposed using GPS trajectories; (3) The discussion of the relationship between the accuracy of the cleaned GPS data and the similarity threshold provides a possible way to extract GPS data based on the estimation accuracy; (4) The amount of GPS data can be compressed after data cleaning, which can greatly decrease the storage space, as well as the computing time.

## 2. Related Work

Based on previous studies, approaches to GPS time sequence data cleaning can be broadly classified into statistical/quantitative based and logical/constraint based [13,14,15,16]. The statistical/quantitative methods of vehicle GPS data cleaning have been widely applied to identify and clean GPS data as they are less susceptible to error stemming from sampling intervals. For example, a high density of GPS points suggests a high probability that a road is present, whereas a low density indicates that vehicles deviate far away from the road [17,18,19]. Therefore, low-density points are defined as outliers. To remove these outliers, several studies [17] have sorted all the data points in ascending order according to their distance from the median and chose 95% of the sorted data points as the experimental data. Kernel density was applied to compute the density of each GPS point and then all the low-density points were removed [18]. In [19], researchers proposed an adaptive density optimization method to automatically recognize outliers and then removed those outliers. In addition, an RGCPK (region growing clustering with prior knowledge) method was also proposed to delete outliers from raw traces based on the motion tendency of vehicle traces [20]. However, the existing methods proposed in articles [17,18,19,20] still cannot remove outliers mixed in the high-density points cluster. 

Trajectory filtering is a typical example of the logical/constraint methods for GPS data quality management. This method has been applied to improve GPS data position accuracy, including adaptive Kalman filtering for INS/GPS [21] and particle filtering. Authors in [22] argued that they can apply various filtering techniques to a trajectory to smooth the noise and potentially decrease the error in the measurements. They also gave a detailed introduction on how to implement a filtering algorithm to smooth a trajectory using the measuring position, speed, and heading of a GPS tracking point in a trajectory. However, the kinds of methods such as Kalman filter and particle filter have the shortcomings of high complexity and computational overhead [22]. 

Unlike previous approaches that clean GPS trajectories based on clustering or filtering algorithms, we propose a GPS data cleaning approach through the adjustment of movement consistency of GPS data. The following section on the data cleaning model describes our method for raw GPS data cleaning. Subsequent sections discuss the experimental results and conclusions.

## 3. Preliminaries

### 3.1. Spatial Big Data: Vehicle GPS Data

Vehicle GPS data as a major part of spatial big data [23] record the position, gathering time, heading, speed, and other movement attributes of moving objects. In general, the sampling rates of those vehicle trajectories usually range from 1 s to 60 s or even longer. The GPS data cleaning method proposed in this paper focuses on finding high-quality GPS data, also known as high-accuracy GPS data, from the raw GPS database. In reality, the position accuracy of the GPS data is different because of the types of GPS receivers, collection environment, techniques (e.g., single-point positioning, precise point positioning, and difference positioning), etc. For instance, the position accuracy of raw GPS traces collected by taxis using single-point positioning technique is about 10–15 m in Wuhan, while raw GPS data generated by smartphone applications on some mobile phones have a 3–5 m accuracy. Beyond that, the accuracy of GPS data collected by the same GPS receiver also displays a difference in different environments (e.g., open area, semi-sheltered area, and sheltered area) [24,25]. At the same time, because of the influence of the error distribution law of the GPS data [26], the accuracy of each GPS point of the trajectory is likely to be different. For instance, if the accuracy of a GPS dataset is about 10 m, the accuracy of one part of such GPS data is higher than 10 m, while another portion of the GPS data shows a lower accuracy. Therefore, a raw crowdsourcing GPS database has both low-accuracy and high-accuracy GPS data; a trajectory in such a database also has both low-accuracy and high-accuracy GPS data. Although most commercial GPS receivers usually implement strong filtering techniques to obtain very smooth tracking results, a considerable amount of crowdsourced GPS data generated by low-end GPS devices are still spotty.

### 3.2. Discussion: Movement Consistency of Vehicle GPS Data

**Figure 1:**
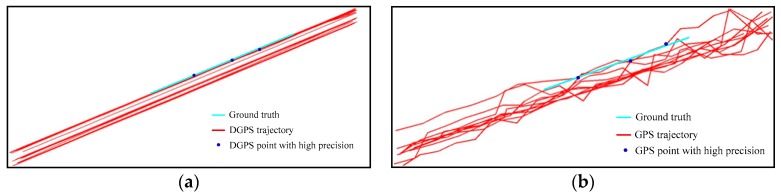
Movement consistency and position accuracy of the GPS data. (**a**) Differential Global Positioning System (DGPS) data overlay with the ground truth; (**b**) GPS data overlay with the ground truth.

The GPS data records the movement of moving objects; the higher the accuracy of the GPS data, the more realistic is the moving pattern it describes. As we know, in the real world, vehicles always keep moving in a straight direction except for changing lanes or turning at intersections. Therefore, trajectories generated by those vehicles show a very smooth result when its accuracy is high, as shown in Figure 1. Figure 1a,b respectively illustrate the DGPS (Differential Global Positioning System) data with 0.1 m accuracy and its synchronous GPS data with 10 m accuracy collected by a mapping car. The model of the GPS receiver and base receiver are Trimble_R9 and NetR9, respectively. The ground truth of one of the trajectories is obtained by field measurements. As we can see from Figure 1a, the DGPS data truly reflect the movement of the mapping car; however, the GPS data cannot paint the true path of the mapping car because of the interference of some low-accuracy GPS points. Meanwhile, by comparing with the ground truth, we found that the positions of GPS points vacillate around the ground truth and some high-accuracy GPS points keep a high consistency in position and direction, as shown in Figure 1a. The DGPS data, by contrast, show a very smooth result, and most of the points are either very near to or are at the ground truth. 

Through the comparative results above, the high-accuracy GPS points of the trajectory present a high consistency of the movement. Based on this observation, the key techniques of GPS data cleaning from the raw crowdsourced database are to construct the consistency model of GPS points based on such consistency of high-accuracy GPS data.

## 4. GPS Data Cleaning Method Based on Movement Consistency

### 4.1. Overview

**Figure 2:**
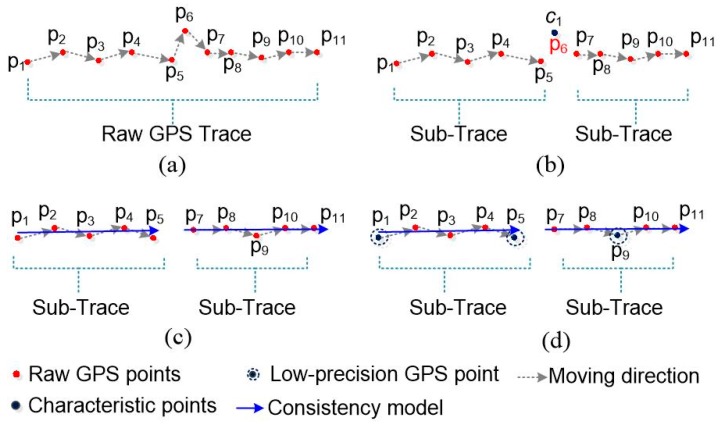
The methodology of GPS data cleaning: (**a**) the raw GPS trace data; (**b**) how the sub-traces are partitioned using a characteristic point *p*_6_; (**c**) the consistency model generated for each sub-trace; and (**d**) the results of cleaning based on the similarity between the consistency model and sub-trace points.

According to the analysis discussed in the previous section, the data cleaning method proposed in this paper has two steps: trajectory segmentation and movement consistency modeling, as shown in Figure 2.

Step 1. The whole trajectory is partitioned into a set of sub-trajectories based on the movement characteristic constraints, as shown in Figure 2a,b. These split points, also called characteristic points, are the starting and ending points of each sub-trajectory.
Step 2. The movement consistency model of each sub-trajectory is constructed using the random sample consensus algorithm based on the high spatial consistency of high-quality GPS data, as shown in Figure 2c,d. The movement consistency model is regarded as the linear position reference for cleaning points; the more similar the GPS points are to the movement consistency model, the more precise are the GPS points.

This section presents a detailed introduction of each process. 

### 4.2. Trajectory Segmentation Based on the Changes in Motion Status of Vehicles

Trajectory segmentation is a preparatory work in spatiotemporal data mining [27]. For example, Gonzales et al. [28] identified critical points in various GPS trajectories to perform their mode classification study. In general, the whole trajectory is divided into several sub-trajectories based on the movement characteristic constraints such as position, time interval, velocity, etc. [29,30]. In this paper, we focus on GPS data cleaning based on the movement consistency. The cleaning rule of the proposed method is based on the premise that a moving object keeps moving on the same road in the same direction. Thus, trajectory segmentation aims to determine the characteristic points where the position or direction of a trajectory changes rapidly and then splits the trajectory base into the detected characteristic points.

#### 4.2.1. The Principle of Trajectory Segmentation 

**Figure 3:**
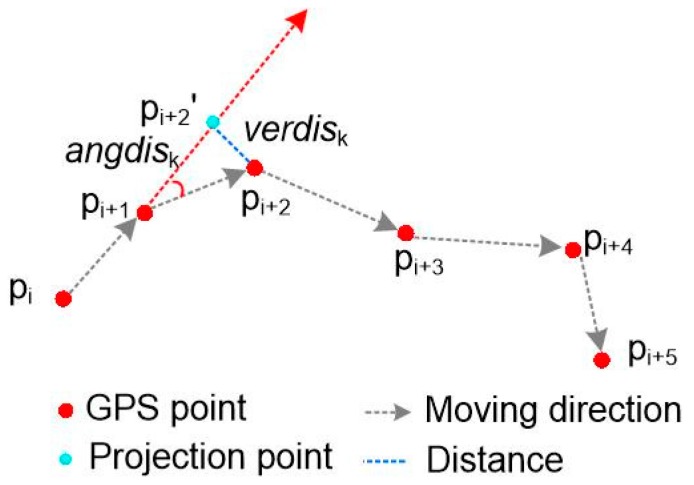
Trajectory partitioning based on position and angle constraints.

The partitioning constraint factors in trajectory segmentation include position and angle, and are termed *verdis*_k_ and *angdis*_k_ in the following definitions:

**Definition 1** (Position interference verdis_k_)**.** *Let T_i_ = (p_1_, p_2_, …, p_n_) denote the trajectory of the object moving from p_1_ to p_n_. For any tracking points p_k_* *∈ T_i_, k = 1, 2, …, n, the vector composed by p_i_ and p_i+1_ presents move action, i = 1, 2, …, n, and p_i+2_′ is the projection of p_i+2_ on the vector of p_i_ and p_i+1_, then the distance between p_i+2_ and p_i+2_′ is called the position interference verdis_k_, as shown in Figure 3.*

**Figure 4:**
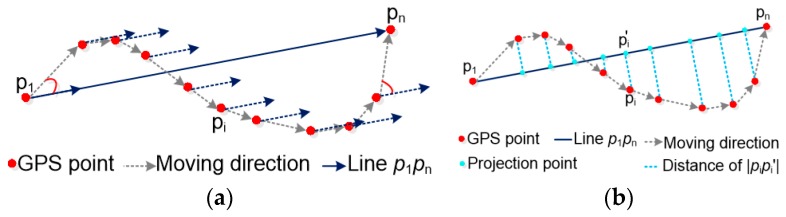
The complexity of trajectory in direction and position.

**Definition 2** (Angle jamming angdis_k_)**.** *Let T_i_ = (p_1_, p_2_, …, p_n_) denote the trajectory of an object moving from p_1_ to p_n_, as shown in Figure 4. For tracking points (p_i+1_, p_i+2_)* *∈ T_i_, i = 1, 2, …, n, the vector composed by p_i+1_ and p_i+2_ is the present movement, the angle between **p_i_p_i+1_** and **p_i+1_p_i+2_** is the angle jamming value angdis_k_, as shown in Figure 3.*

**Definition 3** (Partitioning termination threshold a_1_ and a_2_)**.** The variables a_1_ and a_2_ respectively represent the partitioning termination thresholds in distance and angle.

The main idea of trajectory segmentation for GPS data cleaning is to check the value of *verdis*_k_ and *angdis*_k_, *k* = 1, 2, …, *n*, with respect to the present movement. This algorithm is introduced as follows:

Step 1. input the trajectory *T_i_* (*p*_1_, *p*_2_, *p*_3_, …, *p_n_*);
Step 2. initialize the partitioning parameters’ characteristic points *C*, *c*_1_, *startIndex*, *currIndex*, *length*, *a*_1_, and *a*_2_, and set *c*_1_ = *p*_1_, *startIndex* = 1, *length* = 1;
Step 3. set *currIndex* = *startIndex* + *length*. If *currIndex* < *n*, go to Step 4; otherwise, go to Step 8;
Step 4. set *j* = *startIndex* + 2;
Step 5. calculate *verdis*_j_ and *angdis*_j_. If *verdis*_j_ > *a*_2_ || *angdis*_j_ > *a*_1_, go to Step 6; otherwise, go to Step 3;
Step 6. push *p*_j_ into *C* and set *startIndex* = *j* − 1, *j* = *j* + 1;
Step 7. if *j* < *n*, go to Step 5; otherwise, go to Step 3;
Step 8. push *p_n_* into *C*, and return *C*.

Based on this segmentation algorithm, a trajectory is divided into several sub-trajectories if any one of *verdis*_k_ and *angdis*_k_, *k* = 1, 2, …, *n*, with respect to the present movement meet the partitioning termination thresholds *a*_1_ and *a*_2_. The sub-trajectories will be regarded as the basic unit for the remainder of the cleaning. It should be noted that the characteristic points are stored and managed separately from the sub-trajectories after cleaning since they could be used for trajectory compression or abnormal behavior detection.

#### 4.2.2. Segmentation Threshold Determination 

The distance and angle thresholds (*a*_1_ and *a*_2_) are used to determine whether the tracking point has departed from the centerline of the original route. In general, a GPS point is considered as a turning point if the vertical distance and angle between its two adjacent GPS vectors exceed the maximum width of the road or the minimal angle of the traffic turn in a city. These turning points could be considered as characteristic points that indicate the moving object has changed the moving route or direction. Beyond that, for different types of trajectories, different *a*_1_ and *a*_2_ values should be set for trajectory partitioning relative to their different shapes and unique characteristics. For a trajectory, the more complicated the shape, the more characteristic points are found in that partition. Thus, this study defines two deciding factors to determine the partitioning termination threshold for each trajectory. Especially, the first deciding factor is a global range of distance and angle in trajectory partitioning for all GPS data; and its value is decided by the knowledge of traffic law in a city. The second deciding factor is determined by the shape complexity of a trajectory. Both of those factors combine to determine a specific partitioning termination threshold for each trajectory as follows:

(1) $$a_{1}=\lambda_{1}+g(\beta_{1})$$

(2) $$a_{2}=\lambda_{2}+g(\beta_{2})$$

where *λ*_1_ and *λ*_2_ are the variables of the first deciding factor in the aspects of distance and angle, respectively. In our study, the values of *λ*_1_ and *λ*_2_ equate with the maximum width of the road and the minimum angle of the traffic turn in a city, respectively. The functions *g*(*β*_1_) and *g*(*β*_2_) demonstrate the relationship between the partitioning scale and the shape complexity of trajectories in the aspects of distance and angle, respectively. The variables *β*_1_ and *β*_2_ represent the shape complexity of a trajectory in aspects of distance and angle, respectively. 

Given the significant inverse correlation between the shape complexity and the partitioning termination threshold of a trajectory, the higher the values of *β*_1_ and *β*_2_, and the lower the values of *g*(*β*_1_) and *g*(*β*_2_). Furthermore, the values of the partitioning termination thresholds *g*(*β*_1_) and *g*(*β*_2_) must be sensitive to the shape complexity *β*_1_ and *β*_2_ of trajectories within the set range. When the values of the shape complexity *β*_1_ and *β*_2_ of a trajectory are over a set range, then the partitioning thresholds *g*(*β*_1_) and *g*(*β*_2_) have less variation in distance and angle thresholds, respectively. Therefore, on this basis, combining the previous work for estimating the shape complexity of trajectories [31,32], the logarithmic function of the elementary function is used to model the relationship of *β*_1_ and *g*(*β*_1_) and *β*_2_ and *g*(*β*_2_) as follows:

(3) $$g\left(\beta_{1}\right)=\log_{a}{}^{\beta_{1}}$$

(4) $$g\left(\beta_{2}\right)=\log_{a}^{\beta_{2}}$$

where ‘*a*’ is the base number of functions *g*(*β*_1_) and *g*(*β*_2_), 0 < *a* < 1. To always keep the values of *a*_1_ and *a*_2_ positive, the absolute values of *g*(*β*_1_) and *g*(*β*_2_) must be smaller than the values of *λ*_1_ and *λ*_2_. Based on the research, the movement parameters are usually used to describe the complexity of trajectories [33]. In the movement feature set, classic descriptive statistics of movement parameters, which include the mean, standard deviation, and skewness of moving speed, the turning angle, and straightness index, are extracted from trajectories as basic movement features. In this paper, the standard deviations of projection distance and turning angle are used to represent the complexity of trajectory in position (*β*_1_) and direction (*β*_2_), respectively, as follows:

(5) $$\begin{array}{ll} & \beta_{2}=\sqrt{\frac{1}{n-2}\sum_{i=2}^{n-1}{\left(\left|p_{i}p_{i}^{\prime }\right|-\mu_{dis}\right)}^{2}}\\ where & \\ & \mu_{dis}=\frac{1}{n-2}\sum_{i=2}^{n-1}\left(\left|p_{i}p_{i}^{\prime }\right|\right)\\ & \left|p_{i}p_{i+1}\right|=\sqrt{{\left(x_{i}-x_{i+1}\right)}^{2}+{\left(y_{i}-y_{i+1}\right)}^{2}}\\ & \left|p_{1}p_{n}\right|=\sqrt{{\left(x_{1}-x_{n}\right)}^{2}+{\left(y_{1}-y_{n}\right)}^{2}} \end{array}$$

(6) $$\begin{array}{ll} & \beta_{1}=\sqrt{\frac{1}{n-1}\sum_{i=1}^{n-1}{\left(∠\left(\vec{p_{i}p_{i+1}},\vec{p_{1}p_{n}}\right)-\mu_{ang}\right)}^{2}}\\ where & \\ & \mu_{ang}=\frac{1}{n-1}\sum_{i=1}^{n-1}∠\left(\vec{p_{i}p_{i+1}},\vec{p_{1}p_{n}}\right) \end{array}$$

The complexity of the trajectory is positively associated with the values of *β*_1_ and *β*_2_, and higher values of *β*_1_ and *β*_2_ indicate a higher complexity of the trajectory in distance and angle. As shown in Figure 4, given a trajectory *Tr_i_* = (*p*_1_, *p*_2_, …, *p_n_*), *p_i_* is the tracking point in the trajectory *Tr_i_* and *p_i_* = (*x_i_*, *y_i_*), *i* = 1, 2, …, *n*, *p*_1,_ and *p_n_* are respectively the start and end points of *Tr_i_*. The variables *β*_1_ and *β*_2_ are computed by Equations (5) and (6), where ∠($\underset{p_{i}p_{i+1}}{\to }$, $\underset{p_{1}p_{n}}{\to }$) represents the angle between the vector *p_i_p_i_*_+1_ (denoted as $\underset{p_{i}p_{i+1}}{\to }$) and the vector *p*_1_*p_n_* (denoted as $\underset{p_{1}p_{n}}{\to }$), *i* = 1,2, …, *n*. At the same time, to avoid the extreme values of *β*_1_ and *β*_2_ by a looping trajectory, it is necessary to compare the positions of *p*_1_ and *p_n_* first. If the starting point *p*_1_ overlaps with the ending point *p_n_*, then *p_n_* is replaced by *p_n_*_−1_. This process is repeated from *p_n_* to *p*_2_ until a point is found that does not overlap with the starting point *p*_1_.

### 4.3. GPS Data Cleaning Based on Movement Consistency

#### 4.3.1. The Consistency Model Construction for Each Sub-Trajectory 

**Figure 5:**
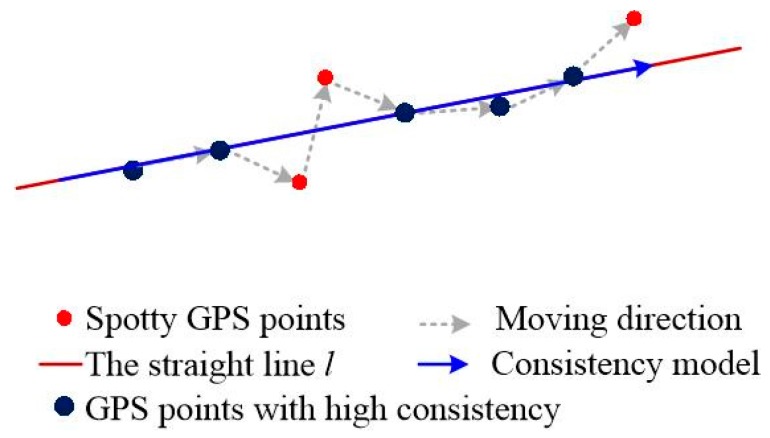
Construction of the consistency model by using RANSAC.

The sub-trajectory reflects the tendency of moving objects as they keep moving on the same road in the same direction. On this basis and combing through the discussions in Section 4.2, we find that high-accuracy vehicle tracking data are highly consistent with position and direction. For instance, tracking points of vehicles with high position accuracy always cluster together along the centerline of each lane, while also having similar headings. Thus, in this paper, we propose using this movement consistency to find high-quality GPS data from the raw GPS database. Specifically, the movement consistency model is defined as a directed line segment that belongs to the straight line *l*, as shown in Figure 5. Since a trajectory has been segmented into a set of sub-trajectories based on trajectory segmentation, GPS tracking points of each sub-trajectory keep with similar headings except for a few curves. Therefore, the construction of movement consistency models of each sub-trajectory equates with the generation of straight line *l*. At present, the least squares method is the most commonly used method for parameter estimation. However, the estimated parameters from a least squares model can be corrupted by outliers. To avoid the effects of these outliers, we use the Random Sample Consensus (RANSAC) algorithm to find GPS points with high consistency in position and heading and then get the consistency model of each sub-trajectory. 

Given a sub-trajectory *STr_i_* = (*p_i_*, *p_i_*_+1_, …, *p_i_*_+*t*_), *p*_k_ = (*x*_k_, *y*_k_), *k* = *i*, *i* + 1, …, *i* + *t*, *STr_i_* ∈ *Tr_i_*, assuming that the consistency model of *STr_i_* belongs to the straight line *l*. Where *x*_0_ and *y*_0_ are the points that go through the consistency model, then *b*_0_ and *b*_1_ are the coefficients of the straight line *l*:

(7) $$\begin{array}{l} x=x_{0}+b_{0}t\\ y=y_{0}+b_{1}t \end{array}$$

The estimated model in the RANSAC algorithm is termed M* and the same as Equation (7). The threshold *τ* defines a GPS tracking point pi and conforms to model M*. The number of iterations is set as *N* and the parameter *s* is used to represent the number of data elements required to fit M*. The concrete procedure for finding position points with high consistency using the RANSAC algorithm was obtained from [34]. 

#### 4.3.2. Discussion of Similarity and Consistency Model for GPS Data Cleaning 

The consistency model of each sub-trajectory is constructed based on the movement consistency of high-accuracy GPS points. Thus, for a sub-trajectory, the value of the similarity between a GPS point and its consistency model relates directly to the level of the position accuracy of it. In this study, the similarity evaluation between a GPS point and the consistency model in distance and direction is defined as Equation (8) by consulting the previous methods [35]:

(8) $$sim_{\left(p_{t},G\right)}=\omega_{1}e^{-\left|p_{t}p_{t}^{\prime }\right|}+\omega_{2}e^{-\left(1-\cos\left(\theta_{t}\right)\right)}$$

where |*p_t_p_t_*′| is the distance between *p_t_* and its projection point *p_t_*′ on the consistency model, *θ_t_* is the angle between *p_t_*′ heading angle and the direction of the consistency model, *ω*_1_ and *ω*_2_ are the weight of the vertical distance and angle, *ω*_1_ + *ω*_2_ = 1. The similarity of GPS measurements and the consistency model range from 0 to 1.

Based on the results of similarity calculation, the high-quality GPS data are detected by setting different similarity thresholds. All cleaned GPS points will be joined back into a long trajectory and be used as raw material for information mining (e.g., road network generation, traffic flow detection, human mobility pattern mining, etc.). The similarity threshold determines the smoothness and quality of cleaned GPS points, and each similarity should correspond to an estimation accuracy of GPS data. However, since there are still many uncertainties in movement consistency construction, it is very difficult to obtain the definite relation between the similarity and the estimation accuracy of GPS data. In this paper, we use the relation of similarity (denoted as *Sim*) and position deviation (denoted as *ε*, also called an estimation accuracy) between GPS data and the ground truth to estimate the similarity threshold. The detailed analysis of the relation between *Sim* and *ε* by using GPS data in the real world is discussed in the next section. 

## 5. Experimental Study 

### 5.1. Experimental Dataset

**Figure 6:**
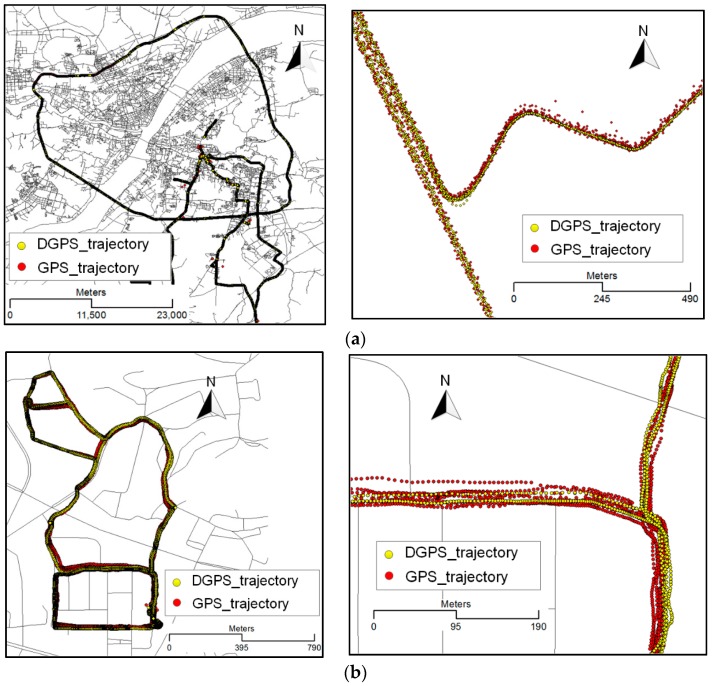
Experimental data. (**a**) DGPS and synchronized GPS trajectories collected by IMU/DGPS systems and GPS loggers; (**b**) DGPS and synchronized GPS trajectories collected by IMU/DGPS systems, smartphones, and hand-GPS.

To test the performance of our method, we experimented with real trajectory datasets. The experimental trajectory data were collected by several shuttle vehicles in Wuhan. These shuttle vehicles were equipped with the GPS logger (model: Trimble_R9), several smartphones (model: MDM6610, UBX-G6010-ST, MTK-MT6627, etc.), hand-GPS (model: SIRF systems), and an inertial measurement unit (model: POS310PCS) that recorded two kinds of traces, GPS and synchronized DGPS traces. It must be stressed that one GPS point corresponds to one DGPS point and all points represent the position of a moving object with different positional accuracies. The position accuracies of the GPS and DGPS data in an urban area were about 10–15 m and 0.05–0.1 m, respectively. The sampling rate for these data was 1 s. The time interval between two adjacent tracking points on a trajectory was not more than 360 s; otherwise, storing the trajectory was restarted from the position where it exceeded the set value. The data collection period for the shuttle vehicles was 7 days. We obtained about 140 million GPS and DGPS points, as shown in Figure 6.

In our study, the highest accuracy of the cleaned data reached the meter level. The position accuracy of trajectories generated by the IMU/DGPS system reached the centimeter level. Therefore, in a follow-up experiment, the synchronized high-accuracy DGPS traces were regarded as ground truth to validate the effectiveness of the proposed method. The raw low-accuracy GPS data will be considered as the experimental data.

### 5.2. Parameters Discussion

**Figure 7:**
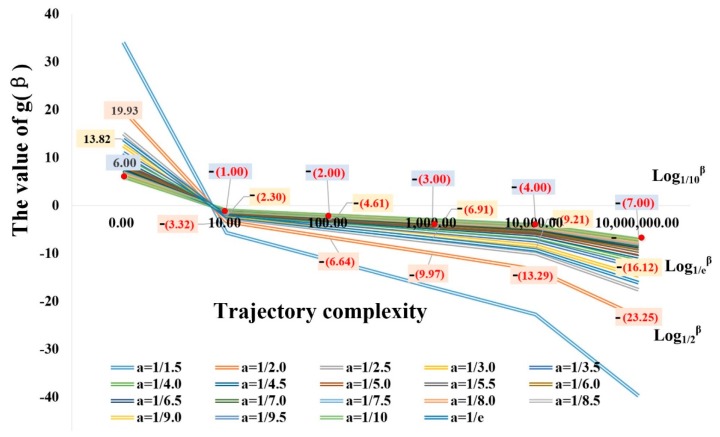
Discussion of base ‘*a*’ in Equation (2).

The constants *λ*_1_ and *λ*_2_, and base ‘*a*’ for partitioning threshold determination are necessary for trajectory partitioning. Based on the above, the value of *λ*_1_ equates with the maximum range of road width and *λ*_2_ depends on the turning angle of vehicles in a city. The experimental traces data were collected in Wuhan. Based on the construction rule of the roads, the maximum width of the one-way road in the experimental region was about 17.5 m, so the value of *λ*_1_ was set as 17.5 m. As the minimum angle of a traffic turn in China is about 60° and the heading error in the GPS data is about 5°–15°, we set the *λ*_2_ to 45°. The value for base ‘*a*’ in Equations (1) and (2) ranges from 0 to 1 and affects the minimum and maximum values of *g*(*β*_1_) and *g*(*β*_2_). Based on Equations (1) and (2), the functions *g*(*β*_1_) and *g*(*β*_2_) have decreasing property with the value of trajectory complexity *β*_1_ and *β*_2_; and are less than zero if *β*_1_ and *β*_2_ are all greater than 1. To always keep the values of *a*_1_ and *a*_2_ as positive, the absolute minimum values of *g*(*β*_1_) and *g*(*β*_2_) must be smaller than the constants *λ*_1_ and *λ*_2_. Figure 7 shows the changing rules of *g*(*β*_1_) and *g*(*β*_2_) with the specific base under different values of *β*_1_ and *β*_2_. The base ‘*a*’ ranges between about 0 and 1.

**Figure 8:**
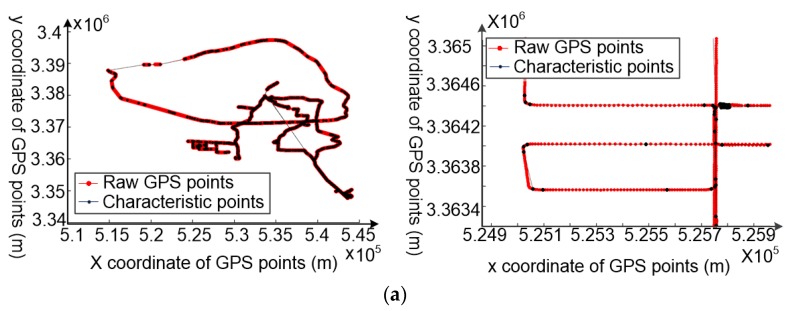

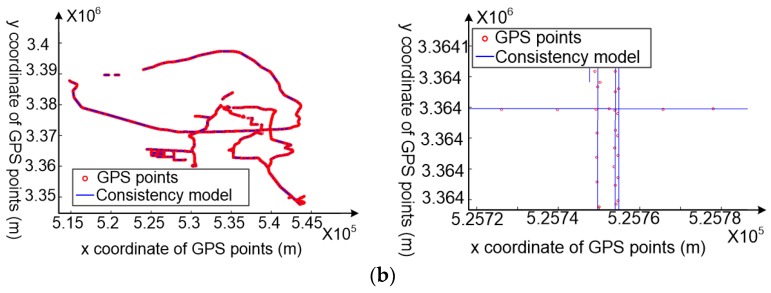
Experimental consistency model construction results for all vehicle movement data. (**a**) The consistency model construction results for the entire dataset; (**b**) the results of a part of the vehicle movement data.

In Figure 7, the smaller the base ‘*a*’, the smaller will be the *g*(*β*_1_) and *g*(*β*_2_) change. As the constants *λ*_1_ and *λ*_2_ are equal to 17.5 m and 45° in this paper, base ‘10^−1^’ was selected as the value of ‘*a*’ in Equation (2). After trace partitioning (Figure 8a), the sub-trajectories are regarded as raw data and cleaned based on the movement consistency model. For consistency model construction, the value of *τ* was set as 0.1 m according to the accuracy requirement; other parameters such as *N* are self-adaptive (Figure 8b). 

A similarity evaluation model is used to calculate the similarity between GPS tracking points and the movement consistency model. This similarity evaluation model is used not only for estimating the similarity of GPS points and the consistency model but for cleaning threshold discussion. These two applications of similarity evaluation model are done to evaluate the similarity between GPS point and high-accuracy spatial reference in aspects of distance and angle. In this paper, the weight of distance and angle of the similarity evaluation model is estimated using the correlation between the distance and angle with measuring errors of GPS data [20]. The experimental results show that the weights in the similarity evaluation model are 0.91 and 0.09, respectively. 

We use the linear regression analysis of the similarity and the position deviation of GPS measurements to derivate the relation of *Sim* and *ε*. With the result of multiple linear regression analysis, the relation of similarity (*Sim*) and position deviation (*ε*) between GPS data and the ground truth fits an exponential model, as shown in Equation (9):

(9) $$Sim=ae^{b\varepsilon }+c$$

**Figure 9:**
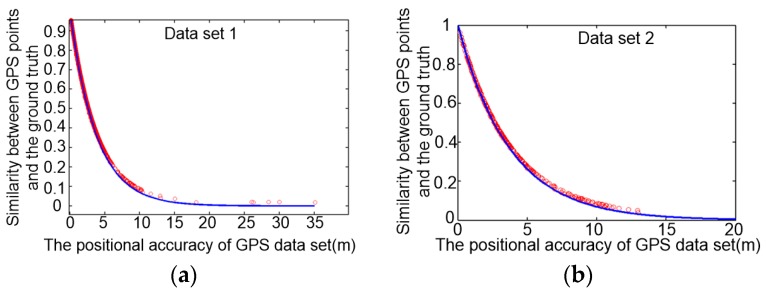
Linear regression results for similarity and position accuracy of the GPS data. (**a**) ‘Dataset 1’ (7604 GPS points) was collected in an urban area on a shadowed road; (**b**) ‘Dataset 2’ (6543 GPS points) was collected in an urban area on a semi-shadowed road.

**Figure 10:**
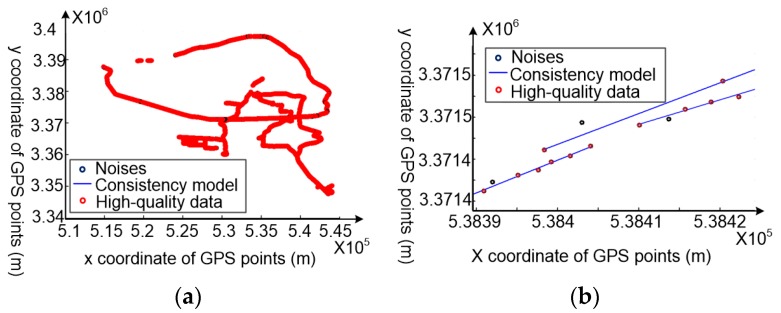
The cleaning results of all vehicle movement data at an estimation accuracy of 3 m. (**a**) The results of the entire dataset; (**b**) the results of part of the vehicle movement data.

The values of parameters *a*, *b*, *c* in Equation (9) are determined by weights of the similarity evaluation model. The cleaning threshold with the specific estimation accuracy is obtained based on Equation (9). Based on plenty of experiment data and analyzing results, the correlation coefficient *R* for *Sim* and *ε* is about 0.942 when the values of *a*, *b*, *c* in Equation (9) for GPS data with 10–15 m accuracy are set as 1, −0.263, 0, respectively. Figure 9 shows the result of exponential regression of similarity and position accuracy of two different datasets collected in different environments with the same overall position accuracy. ‘Dataset 1’ and ‘Dataset 2’ were collected in an urban area on a shadowed road and a semi-shadowed road, respectively. The model of GPS receivers for collecting ‘Dataset 1’ and ‘Dataset 2’ were Trimble R9 and SIRF systems, respectively. The ground truths of these two datasets were obtained based on the CORS system by assembling the GPS receivers and CORS system together. Based on the similarity of Equation (9), we can get some cleaning thresholds by tuning the value of *ε*. Figure 10 shows the cleaned results of GPS points from raw GPS traces of two datasets, with its estimation accuracy set as 3 m; that is, *ε* equals to 3 m. 

### 5.3. Quantitative Evaluation and Discussion

**Figure 11:**
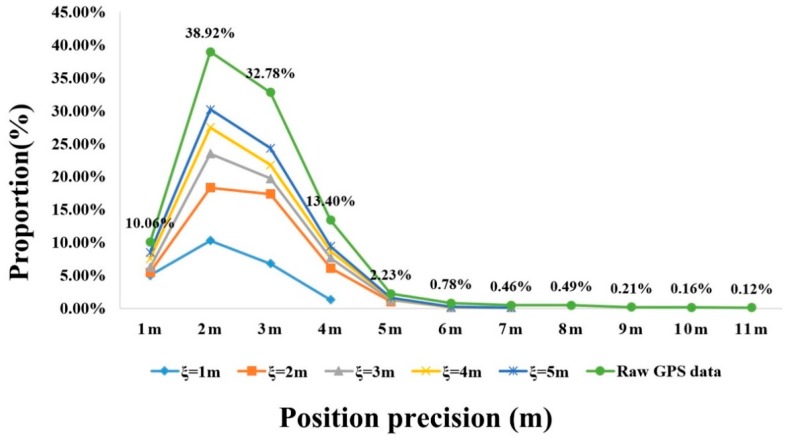
Comparison of the position accuracy of the cleaned data and raw GPS data in different estimation accuracy levels.

To evaluate the effectiveness of the proposed method, we implemented it on the vehicle movement datasets collected in the real world. The position accuracy of those raw GPS data sets is different since the performance of the GPS devices varies. Based on field testing, the average value of the position accuracy of vehicle trajectories collected by Trimble R9, hand-held GPS, and smartphones are about 5.1 m (4.1), 5.0 m (3.6), and 9.1 m (4.7), respectively. The numerical values in parentheses are the standard deviations of each category. The raw datasets were then cleaned depending on different cleaning thresholds that were determined by the values of estimation accuracy. The experimental results for three different GPS datasets are displayed in Table 1. According to the figures given by Table 1, the accuracy and size of the cleaned GPS data are improved greatly compared with the accuracy of the raw dataset, though there is still a difference between the estimation accuracy and the real accuracy of the cleaned GPS data. In addition, based on the experimental results of three tested datasets, the accuracy of the cleaned GPS data also depends on the accuracy of the raw dataset itself. It is still a challenge for us to identify the high-accuracy GPS data from the raw datasets if there is no high-accuracy GPS data in the data in the first place. To further illustrate this point, we analyzed the distribution of accuracy for the cleaned data extracted from the vehicle trajectories collected by Trimble R9, as shown in Figure 11.

In Figure 11, the thick green solid line represents the proportion of raw GPS data in several ranges of position accuracy; the other solid lines show the proportion of cleaned data with different estimation accuracies. We observe that the proportion of GPS points that satisfy changing demands for position accuracy generally increase as the estimation accuracy falls. Although the average value and standard deviation of cleaned data based on the estimation accuracy illustrate that the proposed method is effective, a small percentage of low-position accuracy points beyond the estimation accuracy still exists in the cleaned dataset. For example, a very small subset of GPS points with 4 m accuracy is still mixed in the cleaned data when the estimation accuracy is set to about 1 m. Experimental results demonstrate that it is very difficult to find GPS data at 1 m position accuracy. The reason why the proposed method cannot strictly identify data based on the estimation accuracy is complex. The most important issue is that the GPS error follows a stable distribution; raw GPS points of a sub-trajectory include some high-accuracy points and low-accuracy points. The consistency model constructed using the RANSAC algorithm is considered as the position reference to identify the accuracy of GPS data but sometimes the position of the consistency model may be wrong, especially when there are only low-accuracy points in the sub-trajectory. In addition, the similarity threshold for cleaning is derived from the relation between GPS data and DGPS data, but there are still a lot of uncertainties caused by the collection environment, devices, techniques, etc. In the future work, we will address this problem.

To evaluate the performance of the proposed method, we conducted a qualitative comparison of position accuracy for cleaned data based on the methods discussed in the related work section (e.g., the RGCPK [20], the ADOM [19], the Kernel density method [18], and the Kalman filtering method [17]) and our method. These comparisons of the quality of cleaned data used datasets that were collected by vehicles equipped with Trimble R9. Table 2 shows the highest position accuracy results for the cleaned data from the test datasets using these methods.

According to these results, the datasets employing the method proposed in this paper achieved the highest extracting accuracy when compared to the four other methods. Although RGCPK can also extract high-accuracy GPS data from the raw dataset, the results using RGCPK required prior knowledge to calculate the clustering threshold and filtering standard [20]. The comparison experiment also shows that methods such as ADOM and KDE (Kernel density method) can only remove low-density GPS points. However, sometimes the low-density GPS points do not equal the low-accuracy GPS points, so the cleaning effect is limited [18,19]. The KF (Kalman filtering method) is effective when the trajectory data are particularly noisy [17]. It is usually used to correct GPS data rather than to extract high-quality GPS data from raw datasets. Thus, the accuracy of the cleaned data derived using the filtering method was lowest in comparison with the other methods. Analyzing from practical applications (e.g., road network generation), the high-quality GPS data found from the raw datasets by using our method not only improves the position accuracy of road network extraction results but can also be used to detect lane-based road information.

## 6. Conclusions

Nowadays, the growing volume of spatial big data not only creates process management difficulties but also adds uncertainty for knowledge mining. Unlike previous approaches that clean GPS data based on clustering or filtering algorithms, in this paper, we proposed a method to clean GPS data through the adjustment of movement consistency of GPS data. The mechanism of vehicle GPS data cleaning based on movement consistency includes two steps: trajectory segmentation and consistency model construction. First, the whole trajectory is partitioned into a set of sub-trajectories by characteristic points. Those characteristic points are extracted from trajectories based on the constraints of moving distance or direction. Then, GPS data are cleaned based on the similarities of GPS points and the movement consistency model of the sub-trajectory. The movement consistency model is built using the random sample consensus algorithm based on the high spatial consistency of high-quality GPS data. Moreover, the accuracy of cleaned data can be controlled by tuning the threshold of similarities of GPS data and the local consistency model. The proposed method was evaluated based on extensive experiments, using GPS trajectories generated by a sample of vehicles over a 7-day period in Wuhan, China. Although these experimental results show the effectiveness and efficiency of the proposed method, there are still many problems and shortcomings that need further improving and refining. Due to the position accuracy of the raw GPS data being too low, the proposed method cannot find enough high-quality data from the original database according to the cleaning threshold, which is calculated by the estimation accuracy. In addition, in this paper, GPS data were collected with a high sampling rate by testing vehicles. In the real world, however, the sampling rate of most GPS data is not very high. Thus, this kind of sparse dataset also brings difficulty for data cleaning. In future work, we will address these shortcomings and continue to improve the filtering method proposed here.

## Figures and Tables

**Table 1:** Evaluation of the cleaned data from different datasets.

Trajectory Acquisition Device	Estimation Accuracy: *ε* (m)	Proportion of the Cleaned Data (%)	The Accuracy of GPS Data after Cleaning (Average Value/m)	The Accuracy of GPS Data after Cleaning (Standard Deviation/m)
Trimble R9	2	46.62	2.1	1.0
	3	58.87	2.9	1.2
	4	66.30	3.5	1.8
	5	72.48	4.1	1.8
Hand-held GPS	2	36.86	2.0	0.8
	3	41.38	2.4	1.2
	4	46.32	2.9	1.3
	5	48.76	3.7	2.3
Smartphones	2	27.43	3.8	2.4
	3	32.69	4.8	2.9
	4	40.23	5.1	3.0
	5	48.11	5.6	3.3

**Table 2:** Comparisons of the previous methods for GPS data cleaning.

Methods for GPS Data Cleaning	Vehicle Trajectories Collected by Trimble R9	
	Mean Value of the Accuracy of the Cleaned Data (m)	Standard Deviation of the Accuracy of the Cleaned Data (m)
Method proposed in this paper	2.1	1.0
RGCPK	2.5	1.2
ADOM	4.5	3.2
KDE	4.6	3.3
KF	3.8	7.8
